# Supplementary material for: Tumor Location and a Tumor Volume over 2.8 cc Predict the Prognosis for Japanese Localized Prostate Cancer
Source: Cancers (Basel). 2022 Nov 25;14(23):5823. doi: 10.3390/cancers14235823 (PMC9740872; doi:10.3390/cancers14235823)
Supplement: Supplementary file 1 [file cancers-14-05823-s001.zip › cancers-2046482-supplementary.pdf]

cancers

Supplementary

The Location of Tumor Volume Over 2.8cc Predict the Prognosis Among Japanese Localized Prostate Cancer

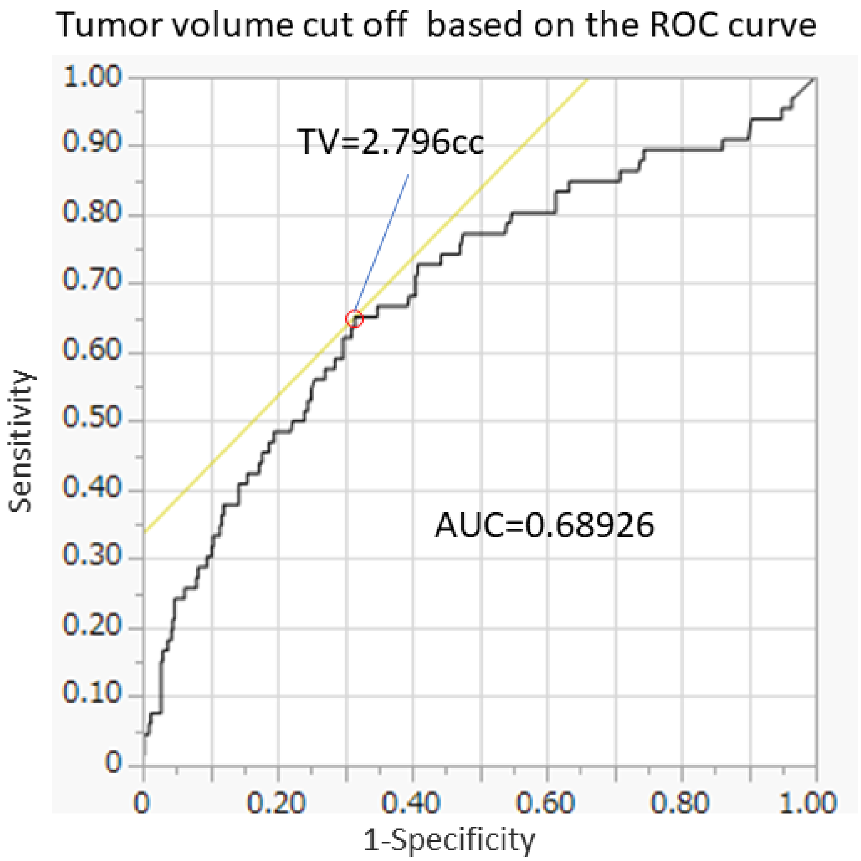

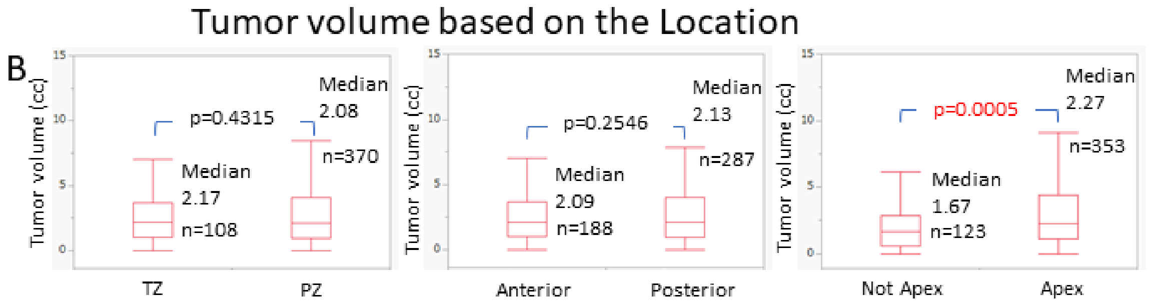

Figure S1. (**A**) The ROC curve was used to calculate AUC, and the optimal cutoff value of tumor volume 2.8cc was obtained. (**B**) Tumor volume based on the location. From the distribution of tumor volume at different prostate locations, the tumor volume at the apex of the prostate was significantly larger than that at the non-apex of the prostate ($p = 0.0005$). There was no significant difference in tumor volume in other parts.

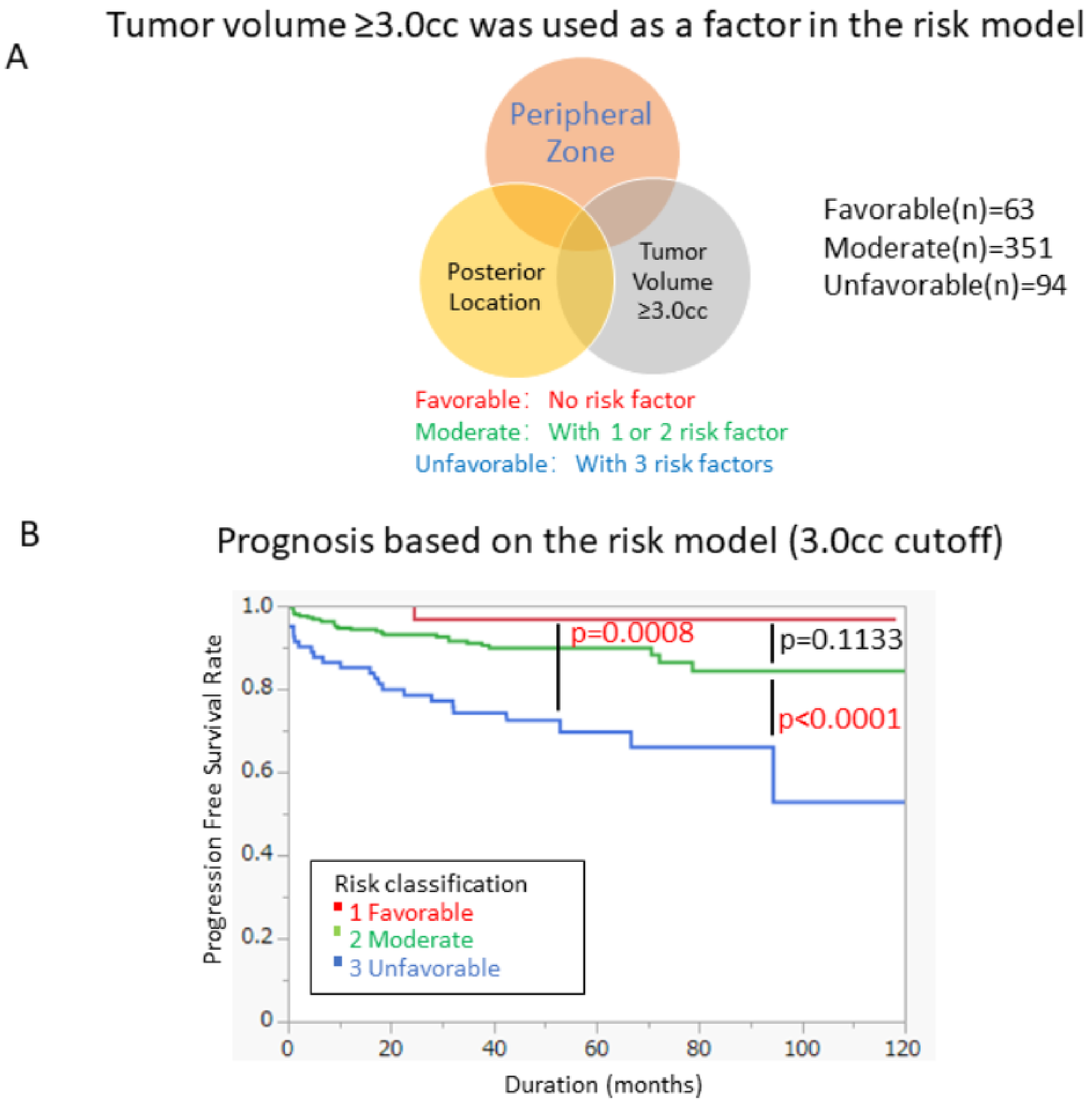

Figure S2. (**A**) A supplemental model that included the 3.0cc tumor volume as one of the factors in the risk model. (**B**) Risk classification significantly differentiated the PFS between the Favorable and Unfavorable group ($p = 0.0008$) and the Moderate and Unfavorable group ($p < 0.0001$).

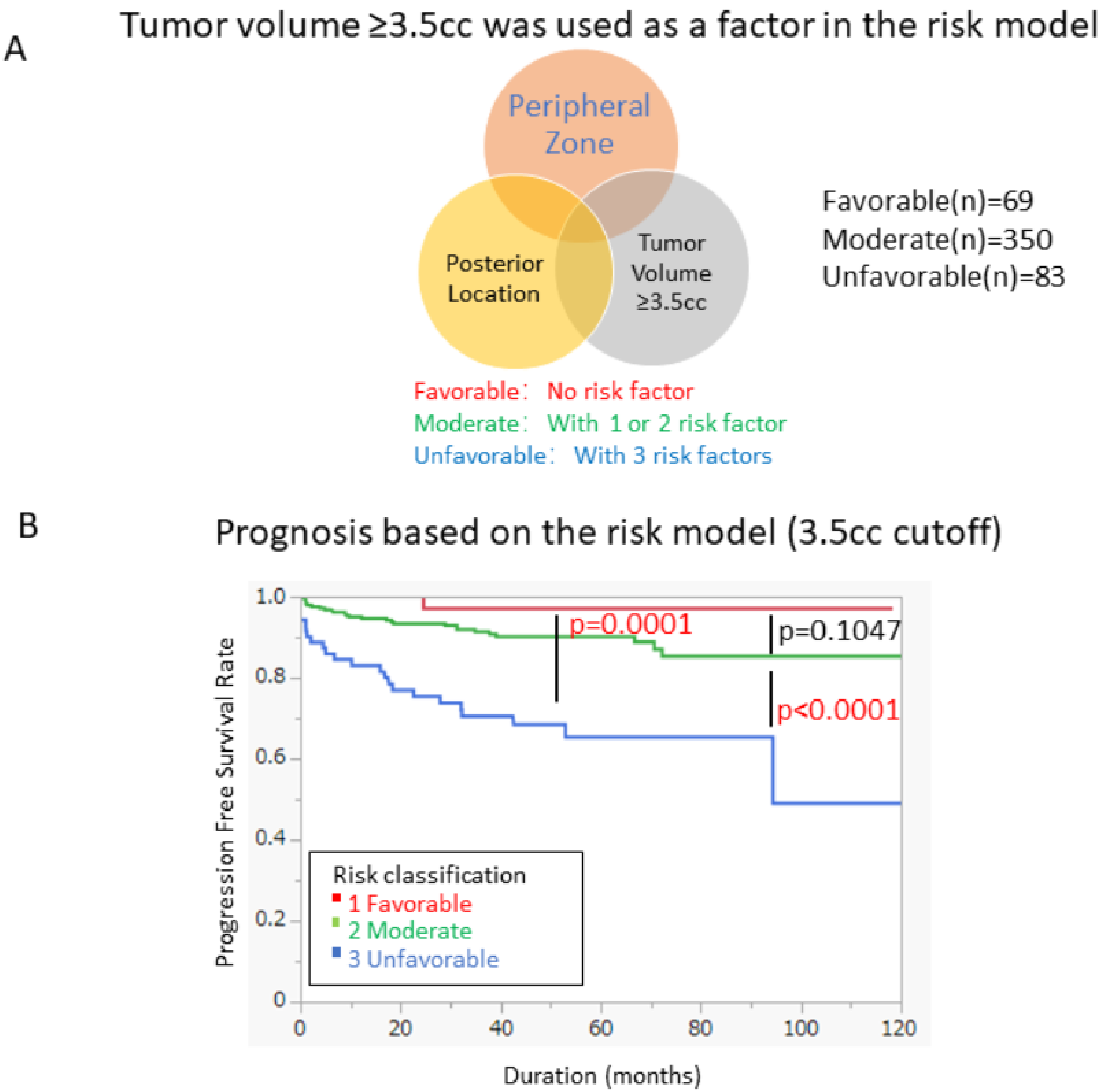

Figure S3. (**A**) A supplemental model that included the 3.5cc tumor volume as one of the factors in the risk model. (**B**) Risk classification significantly differentiated the PFS between the Favorable and Unfavorable group ($p = 0.0001$) and the Moderate and Unfavorable group ($p < 0.0001$).
